# Concerns with AED Conversion: Comparison of Patient and Physician Perspectives

**DOI:** 10.2174/157015909788848947

**Published:** 2009-06

**Authors:** Brien J Smith, Erik K St. Louis, John M Stern, Chad Green, Thomas Bramley

**Affiliations:** 1 Henry Ford Hospital, Detroit, Michigan, USA; 2 Mayo Clinic, Rochester, Minnesota, USA; 3 Geffen School of Medicine at UCLA, Los Angeles, California, USA; 4 Xcenda, Palm Harbor, Florida, USA; 5 Xcenda, Salt Lake City, Utah, USA

**Keywords:** Epilepsy, antiepileptic drugs, conversion, patient preferences.

## Abstract

When discussing AED conversion in the clinic, both the patient and physician perspectives on the goals and risks of this change are important to consider. To identify patient-reported and clinician-perceived concerns, a panel of epilepsy specialists was questioned about the topics discussed with patients and the clinician’s perspective of patient concerns. Findings of a literature review of articles that report patient-expressed concerns regarding their epilepsy and treatment were also reviewed. Results showed that the specialist panel appropriately identified patient-reported concerns of driving ability, medication cost, seizure control, and medication side effects. Additionally, patient-reported concerns of independence, employment issues, social stigma, medication dependence, and undesirable cognitive effects are important to address when considering and initiating AED conversion.

## INTRODUCTION

The objective of an antiepileptic drug (AED) conversion is to improve a patient’s quality of life (QoL). Successful conversion from one AED to another requires effective communication between the clinician and patient. Active patient involvement in the conversion decision, along with well-defined goals, will help to facilitate conversion. However, defining these goals may be difficult if the patient and the clinician have different perceptions and definitions of “success.” Patients with epilepsy are concerned with seizure control, social stigma, adverse effects of medication, and impairment of their cognitive ability [11]. While clinicians share these concerns, they may judge their relative importance differently compared to the patient perspective. For example, the patient may prioritize the decision on conversion in the order of (low cost>seizure control>side effects), compared to the treating physician that may emphasize (seizure control>side effects>cost).

In addition to these varying perspectives regarding the goals of therapy, the patient and clinician will often have different views on the risks of changing therapy. In order for clinicians to facilitate goal attainment by patients, they need to adequately address patient concerns about the AED conversion process. In an effort to identify and compare the perspectives of tertiary referral epilepsy specialists to that of patients, this study reviewed the results of a Delphi panel of epileptologists questioned on their perceptions of patient concerns related to AED conversion. In addition, a literature review of patient-reported concerns regarding epilepsy and AED therapy was conducted.

## METHOD

A Study by Panel of Experts: Considerations for Therapy Replacement in Antiepileptics (SPECTRA) was convened to develop consensus on how to convert patients from one AED to another. To reach consensus, the panel employed the Delphi method, a small-group technique involving a group of geographically dispersed experts [1] answering questionnaires designed to draw out individual responses to the issues posed and to facilitate the refining of views as the group proceeded to agreement through multiple rounds of questioning. The Delphi method avoids the disadvantages of other small-group techniques by maintaining anonymity, controlling feedback, and providing statistically based responses [7].

The literature review was performed using PubMed to identify research publications that addressed patient concerns regarding conversion of AEDs. The literature search identified articles that included the terms “patient” and “epilepsy drug” along with one of the following: “concerns,” “reluctance,” “conversion,” or “perspective.” The search was limited to human subjects and publications in English.

## RESULTS

### Delphi Panel

By questionnaire, panel members were asked to identify topics they discuss with patients in regard to therapy conversion and patient education. Table **1** lists these topics and frequency details. Panel members were also asked to identify concerns that patients express to them with regard to therapy conversion. Table **2** lists these concerns and frequency details.

Panel members were subsequently asked to identify important topics that were missed during the rounds of questioning. Other topics that panel members identified included: teratogenicity in women and adverse effects of both the AED being replaced and the new agent being started.

### Literature Review

A total of 89 articles were identified from the literature review: 44 on patient AND concerns OR reluctance AND epilepsy drug; 18 on patient AND epilepsy drug conversion; 27 on patient perspective AND epilepsy drug. Articles were then reviewed based on relevance to identify patient concerns regarding their epilepsy and AED therapy. A total of nine articles were identified as providing concerns expressed by patients.

**Fig. (1):**
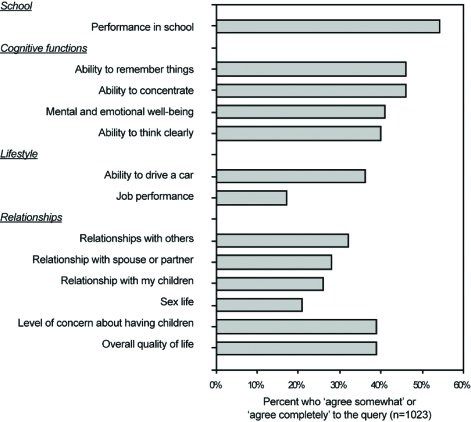
Patient-perceived adverse effects of epilepsy.

**Fig. (2):**
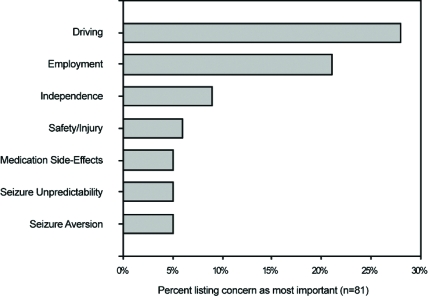
Patient-listed concerns as ‘Most Important’.

**Fig. (3):**
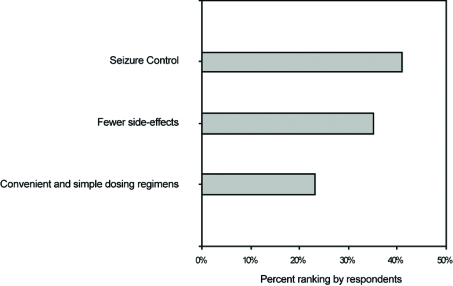
Patient-ranked areas of importance regarding seizure medication.

**Fig. (4):**
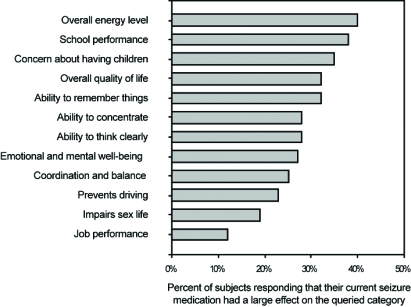
Patient ratings of importance of perceived adverse effects of their current seizure medication.

Of these nine articles, three of them surveyed patient perceptions on several topics [5,6,8]. Select findings of these studies are presented in (Figs. **1** to **4**). Four of the nine identified articles were narrower in scope and focused on a particular patient concern or issue [2-4,10]. Of the two remaining articles, one provided a review of how patients perceive epilepsy and the other described the complex nature of various issues surrounding epilepsy care [9,11].

## DISCUSSION

Successful AED conversion is facilitated by patient acceptance and adherence to therapy goals. In order to effectively perform an AED conversion, patients and clinicians must work together to identify and address patient concerns regarding lifestyle, social, and medical factors. This paper compares patient and physician perceptions on AED conversion. However, the ability to compare current physician perceptions with those of patients from data obtained over the last decade is uncertain and may be limited in light of the significant changes in health care coverage and personal patient cost. 

### Lifestyle and Social Factors

When facing a potential AED conversion, patients may be concerned about such lifestyle and social factors as driving ability, independence, work environment opportunities and prejudices, social stigma, and medication cost. The SPECTRA panel specified the loss or suspension of driving privileges as high on the list of patient concerns regarding AED conversion. Most countries impose driving restrictions upon people with epilepsy due to safety concerns [3]. Specific restrictions on driving vary substantially among jurisdictions and are limited by available data on public and individual risks [3]. The literature review showed that driving restrictions, risks and regulations were identified as an important lifestyle limitation by 11% to 28% of adults [5-8] and an even larger proportion (30%) of teenagers [2].

The SPECTRA panel felt that cost of medication was a principle patient concern in AED conversion. In contrast to the panel findings, however, the literature indicates that cost may be of lesser concern for patients. In one previous study patients listed cost as last among seven criteria that factor into medication choice, [6] although the ability to compare current physician perceptions with those of patients from this prior data from the last decade is uncertain.

Additional patient concerns regarding independence, work environment issues, or social stigma were frequently identified in the literature, indicating that these topics are of high concern to patients. Independence was identified as a concern by 54% of patients, with 9% stating that it was their most important concern [8]. Employment difficulties were reported by 8% of adults and 17% of those in school [5]. Employment was the most important concern for 21% of adult patients [8]. Education and career choices/opportunities were identified as a concern to 60% of teenage patients with epilepsy [2]. Social stigma was a concern for 24% of patients while embarrassment was a concern for 36% of patients [5,8]. Fear of other people’s reactions, shame, and withdrawal from social interactions may lead to isolation and loneliness. As a result, these patient concerns may limit social integration and lifestyle behaviors. Researchers find that a patient’s QoL is adversely impacted when personal development, self-esteem, and compromised relationships are impacted by stigmatization [11]. By addressing concerns about independence, employment, and social stigma with the patient, the health care provider can enhance the AED conversion process by allaying, or at least addressing, patient fears on how the medication conversion may impact their lives.

### Medical Therapy Concerns

From the patient’s perspective, medical therapy concerns include seizure control, medication side effects, medication dependence/safety issues, and cognitive effects. The SPECTRA panel identified patient concerns as potential loss of seizure control, side effects, drug interactions, and dosing frequency. The panel members’ perception regarding the importance patients place on seizure control and side effects is substantiated by literature reports of patient concerns.

Seizure control was ranked as the highest area of importance by 41% of patients with epilepsy [6]. Seizure unpredictability was identified as the most important patient concern by 5% of patients, and another 5% listed seizure aversion as the most important concern [8]. Fear as to when the next seizure may occur was reported by 32% of patients [5]. Patients reported that the single worst medicine outcome would be to make the seizures worse, with 91% of patients resisting medication change if there was a 1% chance of occurrence [6].

Side effects of AEDs were identified as the most important concern by 5% to 35% of patients [6,8]. Teenage patients listed a concern regarding side effects and the possibility of withdrawing antiepileptic medication at 33% [2].

Medication dependence was a concern for 33% of patients, and 31% of patients were concerned with safety [8]. Patients believe that generic substitution can have negative results (68%) and that they are uncomfortable with generic antiepileptic medications (58%) [10]. Among adult patients, 39% reported concern about their children having epilepsy or birth defects [5]. Among teenagers with epilepsy, 14% were concerned with contraception, pregnancy, and the inheritance of epilepsy [2]. Drug interactions were not identified as a topic of patient concern in the literature, but convenience and dosage regimen simplicity was a concern for 23% of patients [6].

The SPECTRA panel did not mention patient concerns associated with undesirable cognitive effects of AEDs. Concerns with cognitive effects are reported by 40% to 50% of patients in school [5]. Improvements in cognition have been reported in QoL when patients are converted to monotherapy [4].

Although this paper attempts to make an accurate analysis of both patient and physician perspectives concerning the AED conversion process this paper has limitations including the design of the initial physician survey, the length of time needed for completion, and the relatively limited data obtained from the literature review that actually addresses patients’ concerns with AED conversion. Also the SPECTRA panel was completed at a time prior to the present concerns with conversion to AED generic preparations.

## CONCLUSION

Understanding patient concerns about epilepsy and AEDs may be important to routine care when a change in medications is being considered or when the patient experiences a problem during AED conversion. Patients’ AED conversion concerns can be lessened by addressing lifestyle, social, and medical factors. Lifestyle and social factors include: driving ability, independence, work/school environment, social stigma, and costs. Medical factors include: seizure control, side effects, dependence/safety, and cognitive effects. Clinician awareness of patient concerns will improve the AED conversion process. Patient education regarding the beneficial impact of therapy conversion will enhance expectations on medical outcomes, allay fears on social impact, enhance compliance, and improve QoL. A clinical approach that stresses caution and explains potential risks and benefits prior to conversion, whether the conversion involves a new AED or change to a generic formulation, should be considered standard clinical practice.

## Figures and Tables

**Table 1:** Topics Reported by the SPECTRA Panel as Discussed with Patients

Topics	n* (%)
Goals of therapy (best QoL, no seizures, no side effects)	12 (100)
Likelihood of having a seizure during conversion	11 (92)
Education regarding difference between short- and long-term side effects	11 (92)
Importance of compliance during therapy conversion	9 (75)
Need to suspend driving	9 (75)

* n = number of individual panel members from the total panel size of 12 specialists who discussed the topic with patients.

**Table 2:** Patient Concerns as Reported by the SPECTRA Panel

Patient Concerns About Conversion	n* (%)
Cost of medication	11 (92)
Loss/suspension of driving privilege	10 (83)
Potential loss of seizure control	10 (83)
New or worsening side effects	10 (83)
Drug interactions	8 (67)
Frequency of dosing	7 (58)

* n = number of individual panel members from the total panel size of 12 specialists who reported patient concerns
